# Gender Specific Association of Serum Leptin and Insulinemic Indices with Nonalcoholic Fatty Liver Disease in Prediabetic Subjects

**DOI:** 10.1371/journal.pone.0142165

**Published:** 2015-11-16

**Authors:** Israt Ara Hossain, Salima Akter, Mohammad Khalilur Rahman, Liaquat Ali

**Affiliations:** 1 Department of Biochemistry and Cell Biology, Bangladesh University of Health Sciences, Dhaka, Bangladesh; 2 Department of Biochemistry and Molecular Biology, University of Dhaka, Dhaka, Bangladesh; University of Warwick – Medical School, UNITED KINGDOM

## Abstract

Adipose tissue-derived hormone leptin plays a functional role in glucose tolerance through its effects on insulin secretion and insulin sensitivity which also represent the risk factors for nonalcoholic fatty liver disease (NAFLD). The present study explored the gender specific association of serum leptin and insulinemic indices with NAFLD in Bangladeshi prediabetic subjects. Under a cross-sectional analytical design a total of 110 ultrasound examined prediabetic subjects, aged 25–68 years consisting of 57.3% male (55.6% non NAFLD and 44.4% NAFLD) and 42.7% female (57.4% non NAFLD and 42.6% NAFLD), were investigated. Insulin secretory function (HOMA%B) and insulin sensitivity (HOMA%S) were calculated from homeostasis model assessment (HOMA). Serum leptin showed significant positive correlation with fasting insulin (r = 0.530, *P* = 0.004), postprandial insulin (r = 0.384, *P* = 0.042) and HOMA-IR (r = 0.541, *P* = 0.003) as well as significant negative correlation with HOMA%S (r = -0.388, *P* = 0.046) and HOMA%B (r = -0.356, *P* = 0.039) in male prediabetic subjects with NAFLD. In multiple linear regression analysis, log transformed leptin showed significant positive association with HOMA-IR (β = 0.706, *P* <0.001) after adjusting the effects of body mass index (BMI), triglyceride (TG) and HOMA%B in male subjects with NAFLD. In binary logistic regression analysis, only log leptin [OR 1.29 95% (C.I) (1.11–1.51), *P* = 0.001] in male subjects as well as HOMA%B [OR 0.94 95% (C.I) (0.89–0.98), *P* = 0.012], HOMA-IR [OR 3.30 95% (C.I) (0.99–10.95), *P* = 0.049] and log leptin [OR 1.10 95% (C.I) (1.01–1.20), *P* = 0.026] in female subjects were found to be independent determinants of NAFLD after adjusting the BMI and TG. Serum leptin seems to have an association with NAFLD both in male and female prediabetic subjects and this association in turn, is mediated by insulin secretory dysfunction and insulin resistance among these subjects.

## Introduction

Nonalcoholic fatty liver disease (NAFLD) is increasingly recognized as a common and potentially severe condition often associated with obesity, type 2 diabetes and hyperlipidemia [1]. However, the pathogenesis of NAFLD remains unclear. Increased synthesis of fatty acids in the liver, increased delivery of free fatty acids to the liver and decreased β-oxidation of free fatty acids may cause the accumulation of fat in the liver. Insulin resistance, through the inhibition of lipid oxidation and increased fatty acid and triglycerides synthesis, is believed to be a key factor in the development of NAFLD. Moreover, insulin resistance states, such as obesity and diabetes, are also characterized by elevated expression and production of several cytokines from the adipose tissue which is considered an endocrine organ regulating body metabolism [2].

Leptin, a 167 amino acid adipocyte derived hormone, has been implicated in the regulation of adipose mass that alter both insulin sensitivity and insulin secretion. Several studies have demonstrated a significant positive correlation between serum leptin and fasting insulin levels that is independent of body adiposity. Leptin has a proinflammatory role and is considered to be an essential mediator of liver fibrosis [3]. Chitturi et al have shown that serum leptin levels are significantly higher in subjects with non-alcoholic steatohepatitis (NASH), when compared to controls [4]. Serum leptin concentration also has a gender dimorphism, with higher serum levels in women than that in men [5]. Segal et al reported that insulin resistance seems to have an association with serum leptin and showed that the serum levels of leptin in insulin- resistant men was higher than that in BMI- matched insulin sensitive men [6]. Serum leptin concentrations are characteristically increased in obese subjects and there appears to be a linear relationship between serum leptin and BMI in women and to a lesser extent in men [7].

It was suggested that insulin resistance to leptin in β-cells in glucose intolerance state, might prevent the inhibitory effect of leptin on insulin secretion resulting in hyperinsulinemia, which might exhaust pancreatic β-cells leading to development of NAFLD. The biological actions of leptin are mediated largely through interaction with its cognate receptor (Ob-RL) expressed in several peripheral tissues including human hepatic cells, which attenuates some insulin-induced activities causing insulin resistance, whereas increased insulin resistance represents an almost universal finding in subjects with NAFLD suggesting a role for leptin [6]. The close relationship of leptin with adipose tissue and fat stores of the body suggests its involvement in the etiology and pathogenesis of NAFLD [8].

Although it is clear that the imbalanced production of pro and inflammatory adipokines contributes to the pathogenesis of NAFLD [9] and their association with diabetes and other obesity induced complications has been suggested [3], however with prediabetes having NAFLD is less clear and also data on its correlation with insulinemic indices are scarce. Hence, we aimed to determine the relationship of circulating serum leptin and insulinemic indices with NAFLD in prediabetic subjects.

## Materials and Methods

### Study population

A total of 110 (one hundred and ten) prediabetic subjects according to the WHO Group Study criteria [10] were purposively investigated who were attending the BIHS Hospital, Darussalam, Dhaka, Bangladesh, to check their glycemic status in the period between March 2012 and October 2013. Subjects suffering from any systemic illness like acute and severe septic conditions, cardiac disease, hepatic, renal, respiratory failure, stroke and type 1 diabetes, those taking drugs that significantly affect the glucose metabolism, antihypertensive, lipid lowering agents and pregnant subjects were excluded. The study was approved from the Ethical Review Committee of Bangladesh Diabetic Association (BADAS). *Ref no*: *BADAS-ERC/13/00106*. Each participant gave written informed consent prior to study inclusion.

### Anthropometric and clinical measurements

Anthropometric measurements were performed by the interviewer, including weight, height, body mass index (BMI) and waist-to-hip circumference. Systolic and diastolic blood pressures were measured by standard clinical procedures. BMI was calculated as weight divided by height squared.

### NAFLD evaluation

Trained ultrasonographer performed liver ultrasound with a 3.5 MHz linear transducer (Philips Ultrasound-Ay-MNT-15 TTK, HDI-4000, Netherland) in fasting state for grading the extent of fatty liver and to look for evidence of portal hypertension. The protocol described by Hamaguchi et al and found 92% sensitivity and 100% specificity for the histological diagnosis of fatty liver [11]. NAFLD was defined as any degree of fatty liver in the absence of alcohol intake. NAFLD, if present, was classified based on standard ultrasonographic criteria as: Grade 1 (mild steatosis): slightly increased liver echogenicity with normal vessels and absent posterior attenuation. Grade 2 (moderate steatosis): moderately increased liver echogenicity with partial dimming of vessels and early posterior attenuation. Grade 3 (severe steatosis): diffusely increased liver echogenicity with absence of visible vessels and heavy posterior attenuation [12].

#### Biochemical analyses

Serum glucose, both at fasting and following ingestion of 75 g of glucose, serum lipid profile [total cholesterol (TC), triglyceride (TG) and high density lipoprotein cholesterol (HDL-c)], liver enzymes like serum glutamate pyruvate transaminase (SGPT), serum glutamate-oxaloacetate transaminase (SGOT), gama glutamyl transaminase (γ-GT) and alkaline phosphatase (ALP) were measured by enzymatic-colorimetric method (Randox, UK) using an automated analyzer (Hitachi 704, Tokyo, Japan). Glycated haemoglobin (HbA_1c_) was measured using the high-performance liquid chromatography (HPLC) method (Variant II, Bio-Rad Laboratories, Hercules, CA, USA). Serum insulin and leptin were measured by an ELISA method (DRG-International, Germany). Low-density lipoprotein cholesterol (LDL-c) was calculated by the Friedewald equation [13]. Insulin resistance (HOMA-IR) was calculated according to HOMA-IR equation = [Fasting plasma glucose (mmol/l) × fasting plasma insulin (μIU/ml)]/22.5 [14]. When specific performance characteristics were assessed for the measurement of serum leptin, the within and between run precision for leptin samples were 5.21% and 8.75% and for serum insulin samples were 5.40% and 7.63% coefficients of variation (CV) respectively.

### Data analysis

Data were expressed as mean ± standard deviation (SD) and/ or number (percentage) where appropriate. Differences between the groups were assessed by Student’s t-test and the association of two parameters was explored by univariate and multivariate analysis as appropriate. Because the distributions of leptin were skewed, we used logarithmically transformed values for statistical analysis. A *P* value less than 0.05 was considered statistically significant. Statistical analyses were performed using statistical package for social science (SPSS) for Windows version 15.0 (SPSS Inc., Chicago, ILL).

## Results

### General characteristics of the study subjects

**Fig 1:**
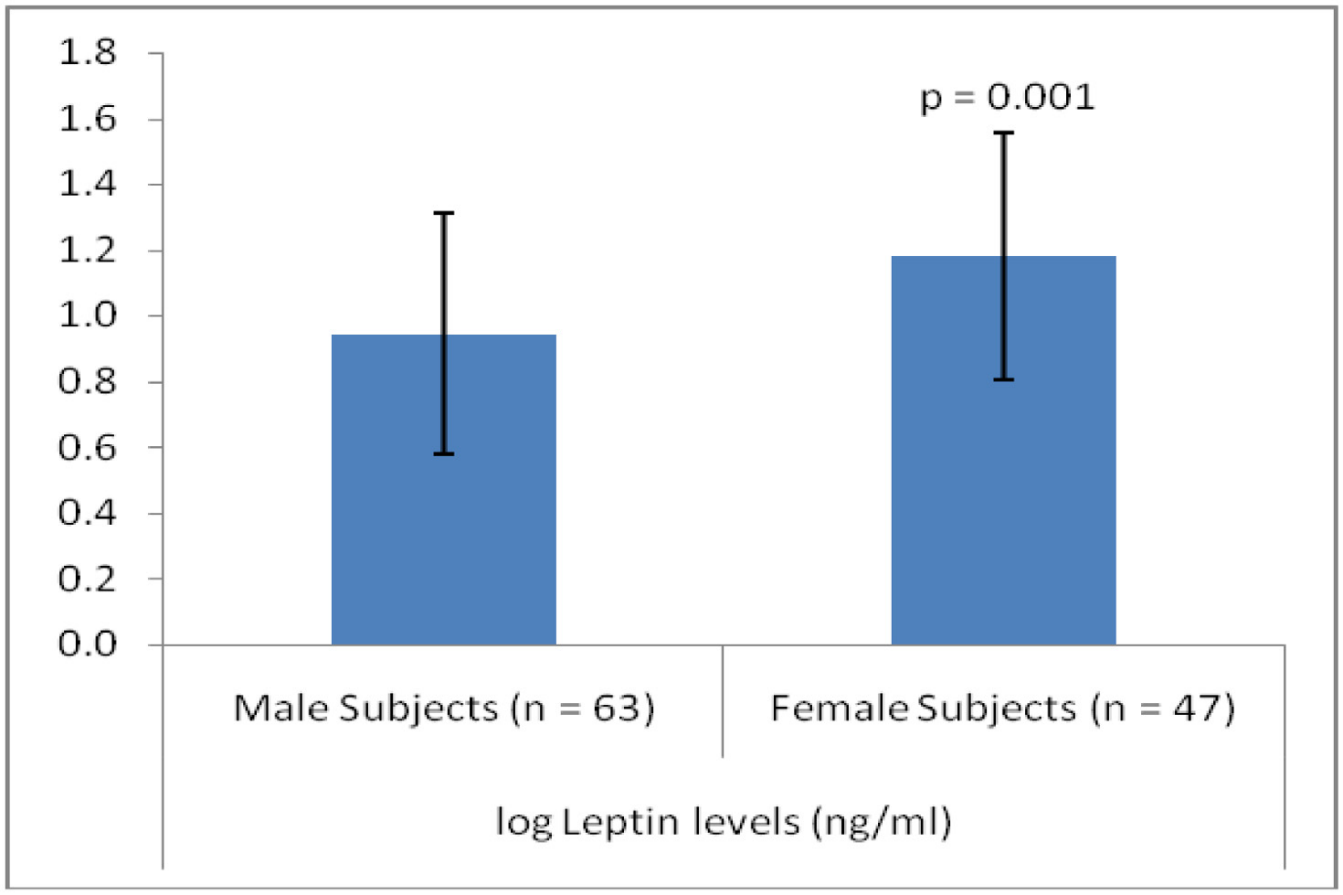
Circulating leptin levels in the study subjects. Log transformed serum leptin was significantly higher in female subjects compared to their male counterparts (*P* = 0.001).

General characteristics of both genders are shown in Table 1. Female subjects had significantly higher levels of BMI, HC, %BF, HDL-c and fasting insulin whereas, male subjects had significantly higher levels of WHR and fasting serum glucose. Log transformed serum leptin was also significantly higher in female subjects compared to their male counterparts (Fig 1).

**Table 1:** General characteristic of the study subjects by gender.

Variables	Male subjects	Female subjects	*P* value
	(n = 63)	(n = 47)	
Age (yrs)	48 ± 8	42 ± 8	< 0.001
Body mass index (kg/m^2^)	24.4 ± 2.7	26.1 ± 5.0	0.029
Waist circumference (cm)	89 ± 7	91 ± 9	0.439
Hip circumference (cm)	94 ± 6	98 ± 9	0.031
Waist to hip ratio	0.95 ± 0.03	0.93 ± 0.05	0.021
Percent body fat	25.6 ± 6.3	35.4 ± 5.6	< 0.001
Systolic blood pressure (mmHg)	122 ± 25	120 ± 28	0.846
Diastolic blood pressure (mmHg)	81 ± 22	84 ± 21	0.568
Subgroups of prediabetes, n (%)			
Impaired fasting glucose (IFG)	30 (47.6)	17 (36.2)	-
Impaired glucose tolerance (IGT)	9 (14.3)	14 (29.8)	-
Combined (IFG-IGT)	24 (38.1)	16 (34.0)	-
Fasting serum glucose (mmol/l)	6.0 ± 0.47	5.7 ± 0.41	0.002
Postprandial serum glucose (mmol/l)	7.8 ± 1.6	8.2 ± 1.5	0.171
Glycosylated hemoglobin (%)	5.7 ± 0.64	5.6 ± 0.55	0.313
Total cholesterol (mg/dl)	190 ± 41	191 ± 40	0.854
Triglycerides (mg/dl)	185 ± 121	158 ± 84	0.169
High density lipoprotein-cholesterol (mg/dl)	34 ± 6	40 ± 8	< 0.001
Low density lipoprotein-cholesterol (mg/dl)	179 ± 42	188 ± 44	0.254
Glutamate pyruvate transaminase (IU/L)	34.9 ± 17.3	32.4 ± 14.2	0.412
Gama glutamate transaminase (IU/L)	30.9 ± 12.4	28.6 ± 15.7	0.431
Alkaline phosphatase (IU/L)	102.7 ± 26.3	108.3 ± 28.5	0.305
Glutamate oxaloacetate transaminase (IU/L)	31.5 ± 19.4	28.0 ± 8.9	0.209
Fasting insulin (μIU/ml)	14.1 ± 5.9	18.5 ± 8.1	0.048
Postprandial insulin (μIU/ml)	60.9 ± 38.1	68.9 ± 45.2	0.321
HOMA%S	56.8 ± 21.9	51.0 ± 23.2	0.185
HOMA%B	121 ± 37	124 ± 34	0.667
HOMA-IR	3.7 ± 1.6	4.1 ± 1.9	0.250
NAFLD evaluation, n (%)			
Grade 0	34 (54)	27 (57.4)	-
Grade 1	25 (39.7)	15 (31.9)	-
Grade 2	2 (3.2)	4 (8.5)	-
Grade 3	2 (3.2)	1 (2.1)	-

Results are expressed as number (percentage), mean ± SD; n = number of subjects; HOMA%B, β cell function assessed by homeostasis model assessment; HOMA%S, insulin sensitivity assessed by homeostasis model assessment; HOMA-IR, insulin resistance assessed by homeostasis model assessment; NAFLD, nonalcoholic fatty liver disease.

### Anthropometric, clinical and biochemical characteristics of both genders according to their NAFLD evaluation

In both genders, WC, SBP, DBP, HbA_1c_, TG, SGOT, fasting insulin, HOMA-IR and leptin were significantly higher as well HOMA%S and HOMA%B were significantly lower in NAFLD subjects compared to their non NAFLD counterparts. In male subjects, BMI, HC, %BF, HDL-c and SGPT and in female subjects, postprandial insulin was significantly higher in NAFLD subjects compared to their non NAFLD counterparts (Table 2).

**Table 2:** Anthropometric, clinical and biochemical characteristics in non NAFLD and NAFLD groups of both genders.

	Male subjects			Female subjects		
Variables	Non NAFLD	NAFLD	*P* value	Non NAFLD	NAFLD	*P* value
	(n = 35)	(n = 28)		(n = 27)	(n = 20)	
Age (yrs)	48 ± 7	47 ± 9	0.688	40 ± 8	43 ± 7	0.228
Body mass index (kg/m^2^)	24 ± 2	25 ± 3	0.045	26 ± 5	27 ± 3	0.399
Waist circumference (cm)	87 ± 6	92 ± 7	0.012	89 ± 9	93 ± 8	0.070
Hip circumference (cm)	93 ± 5	97 ± 6	0.024	97 ± 10	100 ± 8	0.305
Waist to hip ratio	0.9 ± 0.0	0.9 ± 0.0	0.229	0.9 ± 0.0	0.9 ± 0.0	0.176
Percent body fat	24 ± 3	28 ± 8	0.014	34 ± 5	37 ± 5	0.101
Systolic blood pressure (mmHg)	112 ± 17	134 ± 29	< 0.001	112 ± 12	131 ± 39	0.024
Diastolic blood pressure (mmHg)	80 ± 21	88 ± 23	0.059	74 ± 7	97 ± 27	< 0.001
Fasting serum glucose (mmol/l)	6.0 ± 0.4	5.9 ± 0.4	0.377	5.7 ± 0.4	5.6 ± 0.4	0.267
Postprandial serum glucose (mmol/l)	7.5 ± 1.6	8.2 ± 1.5	0.071	8.1 ± 1.5	8.3 ± 1.6	0.654
Glycosylated hemoglobin (%)	5.5 ± 0.6	5.9 ± 0.4	0.003	5.4 ± 0.6	5.8 ± 0.3	0.016
Total cholesterol (mg/dl)	184 ± 40	198 ± 41	0.178	184 ± 36	202 ± 45	0.140
Triglycerides (mg/dl)	160 ± 97	215 ± 14	0.042	138 ± 33	184 ± 119	0.047
High density lipoprotein-c (mg/dl)	36 ± 6	32 ± 5	0.006	42 ± 8	38 ± 8	0.207
Low density lipoprotein-c (mg/dl)	178 ± 35	179 ± 49	0.913	183 ± 47	197 ± 39	0.278
SGPT (IU/L)	28 ± 10	42 ± 21	0.002	32 ± 15	32 ± 13	0.876
GGT (IU/L)	26 ± 9	36 ± 13	0.001	26 ± 17	31 ± 13	0.338
Alkaline phosphatase (IU/L)	100 ± 30	106 ± 19	0.364	110 ± 27	105 ± 30	0.529
SGOT (IU/L)	26 ± 8	38 ± 26	0.011	25 ± 8	31 ± 8	0.042
Fasting insulin (μIU/ml)	12 ± 3	16 ± 7	0.016	13 ± 3	20 ± 10	0.007
Postprandial insulin (μIU/ml)	55 ± 43	67 ± 29	0.225	56 ± 39	85 ± 48	0.030
HOMA%S	63 ± 24	49 ± 16	0.013	58 ± 26	42 ± 14	0.012
HOMA%B	132 ± 40	106 ± 25	0.004	132 ± 27	111 ± 40	0.037
HOMA-IR	3.3 ± .9	4.3 ± 2.1	0.025	3.5 ± 1.0	5.0 ± 2.6	0.013
Log leptin (ng/ml)	0.7 ± 0.3	1.2 ± 0.4	0.001	1.0 ± 0.4	1.4 ± 0.2	0.009

Results are expressed as number (percentage), mean ± SD; n = number of subjects; SGPT, serum glutamate pyruvate transaminase; GGT, gama glutamate transaminase; SGOT, serum glutamate oxaloacetate transaminase; HOMA%B, β cell function assessed by homeostasis model assessment; HOMA%S: insulin sensitivity assessed by homeostasis model assessment; HOMA-IR, insulin resistance assessed by homeostasis model assessment; NAFLD, nonalcoholic fatty liver disease.

### Relationship of log transformed serum leptin with significant variables of both genders according to their NAFLD evaluation

In Pearson’s correlation analysis, WC, HC, %BF and HbA_1c_ showed significant positive correlation with log leptin of both genders having NAFLD. However, male subjects with NAFLD showed significant positive correlation of SGOT, SGPT, fasting insulin, postprandial insulin and HOMA-IR as well as significant negative correlation of HOMA%B and HOMA%S with serum leptin. On the other hand, female subjects with NAFLD only showed significant positive correlation of BMI with serum leptin (Table 3).

**Table 3:** Pearson’s correlation between serum leptin levels and study variables, by gender in NAFLD and non NAFLD groups.

Variables	Male subjects				Female subjects			
	Non NAFLD		NAFLD		Non NAFLD		NAFLD	
	(n = 35)		(n = 28)		(n = 27)		(n = 20)	
	*r*	*P*	*r*	*P*	*r*	*P*	*r*	*P*
Body mass index (kg/m^2^)	0.206	0.236	0.397	0.125	0.117	0.561	0.461	0.048
Waist circumference (cm)	0.239	0.166	0.364	0.043	0.223	0.263	0.392	0.037
Hip circumference (cm)	0.146	0.394	0.380	0.040	0.144	0.475	0.450	0.046
Waist to hip ratio	0.391	0.048	-0.049	0.803	0.132	0.511	-0.132	0.579
Percent body fat	0.129	0.459	0.348	0.043	0.352	0.031	0.361	0.034
HbA_1c_ (%)	0.049	0.781	0.382	0.045	-0.364	0.048	0.209	0.376
HDL-cholesterol (mg/dl)	-0.016	0.929	-0.022	0.913	0.392	0.042	-0.253	0.281
SGOT (IU/L)	-0.261	0.130	0.380	0.040	0.278	0.160	-0.196	0.408
SGPT (IU/L)	0.171	0.326	0.386	0.043	0.364	0.049	-0.091	0.702
Fasting insulin (μIU/ml)	0.119	0.499	0.530	0.004	-0.118	0.559	0.078	0.743
PP insulin (μIU/ml)	-0.056	0.750	0.384	0.042	-0.279	0.159	0.167	0.481
HOMA%S	-0.046	0.797	-0.388	0.046	-0.291	0.142	-0.283	0.226
HOMA%B	-0.205	0.246	-0.356	0.039	0.164	0.415	0.309	0.198
HOMA-IR	0.167	0.339	0.541	0.003	-0.63	0.753	0.045	0.850

Results are expressed as Pearson’s correlation coefficient *r* and statistical significance *P* < 0.05. HbA_1c_, glycated hemoglobin; HDL-c, high density lipoprotein cholesterol; SGOT, serum glutamate-oxaloacetate transaminase; SGPT, serum glutamate-pyruvate transaminase; PP insulin, postprandial insulin; HOMA%S, insulin sensitivity assessed by homeostasis model assessment; HOMA%B, β cell function assessed by homeostasis model assessment; HOMA-IR, insulin resistance assessed by homeostasis model assessment; NAFLD, nonalcoholic fatty liver disease.

### Significant determinant for the association of log leptin with insulinemic indices of both genders according to their NAFLD evaluation after adjusting the effects of major confounders

Multiple linear regression analysis demonstrated that, only male subjects with NAFLD had significant positive association with HOMA-IR after adjusting the effects of major confounders (BMI, TG and HOMA%B) (Table 4). By binary logistic regression analysis, only leptin was found to be significant determinant of NAFLD in male prediabetic subjects after adjusting the effects of potential confounders of BMI, TG, HOMA%B and HOMA-IR respectively. However, leptin, HOMA%B and HOMA-IR were found to be significant determinants of NAFLD in female prediabetic subjects after adjusting the effects of potential confounders of BMI and TG respectively (Table 5).

**Table 4:** Multiple linear regression analysis using log leptin as the dependent variable in both genders.

Non NAFLD group								
Variables	Male subjects				Female subjects			
	β value	*P* value	95% CI		β value	*P* value	95% CI	
			lower	Upper			lower	Upper
BMI (kg/m^2^)	0.255	0.269	-0.724	2.459	0.118	0.682	-0.933	1.382
Triglyceride (mg/dl)	0.220	0.320	-0.084	0.029	0.372	0.186	-0.089	0.414
HOMA%B	-0.175	0.417	-0.115	0.050	-0.405	0.147	-0.066	0.396
HOMA-IR	0.210	0.342	-1.825	5.018	0.153	0.591	-9.279	5.512
**NAFLD group**								
BMI (kg/m^2^)	0.252	0.111	-0.104	0.916	0.574	0.087	-0.488	5.826
Triglyceride (mg/dl)	0.125	0.311	-0.026	0.009	0.083	0.798	-0.078	0.098
HOMA%B	-0.043	0.769	-0.070	0.052	-0.203	0.674	-0.461	0.315
HOMA-IR	0.706	< 0.001	1.147	3.302	0.500	0.249	-4.294	14.360

Dependent variable, serum leptin; Adjusted R^2^, male and female, NAFLD = 0.771 and 0.518; non NAFLD = 0.180 and 0.220; the level of significance at *P* < 0.05; β, regression coefficient; CI: confidence interval; BMI, body mass index; HOMA%B, β cell function assessed by homeostasis model assessment; HOMA-IR, insulin resistance assessed by homeostasis model assessment; NAFLD: nonalcoholic fatty liver disease.

**Table 5:** Binary logistic regression analyses using NAFLD as the dependent variable in both genders after adjusting the major confounders.

Variables	Male subjects					Female subjects				
	Coefficient	*P* value	Odds Ratio	95% CI		Coefficient	*P* value	Odds Ratio	95% CI	
				Lower	Upper				Lower	Upper
BMI (kg/m^2^)	0.106	0.530	1.112	0.798	1.549	-0.033	0.731	0.968	0.802	1.167
TG (mg/dl)	0.007	0.063	1.007	1.000	1.015	0.015	0.278	1.015	0.988	1.043
HOMA%B	-0.023	0.128	0.978	0.949	1.007	-0.060	0.012	0.942	0.898	0.987
HOMA-IR	0.104	0.821	1.110	0.450	2.740	1.195	0.049	3.305	0.997	10.95
Log lep (ng/ml)	0.259	0.001	1.296	1.111	1.512	0.100	0.026	1.106	1.012	1.208

Dependent variable, NAFLD; Adjusted R^2^, male and female = 0.480 and 0.470; the level of significance at *P* < 0.05; BMI, body mass index; TG, triglyceride; HOMA%B, β cell function assessed by homeostasis model assessment; HOMA-IR, insulin resistance assessed by homeostasis model assessment; Log lep, logarithm transformed leptin; NAFLD, nonalcoholic fatty liver disease.

## Discussion

Prevalence of NAFLD has been increasing worldwide and its morbidity and mortality is common in diabetic subjects without any alcohol consumption in South Asian population. In our study, fatty liver among the prediabetic subjects was confirmed by whole abdomen ultrasonography. NAFLD detection by ultrasonography allows reliable and accurate detection of moderate to severe fatty liver, compared to liver biopsy. Though liver biopsy is the gold standard in diagnosing NAFLD and the most accurate tool for grading fibrosis however, is invasive and carries the risk of complications. Bedside ultrasound, as a non-invasive and readily available tool, has an important role in diagnosing NAFLD. Ultrasound is likely the imaging technique of choice for screening for fatty liver in clinical and population settings. Shannon et al found a significant positive correlation between USS and liver biopsy with a Spearman’s coefficient of 0.80 [CI (0.71, 0.88), p value <0.001] [15]. A recent study by Palmentieri et al of 235 patients undergoing US with liver biopsy showed a sensitivity, specificity, PPV, and NPV of 91%, 93%, 89%, and 94%, respectively, for predicting at least 30% steatosis [16].

Several surrogate markers like abnormal liver enzymes have been identified in the pathophysiology of NAFLD. Recently adipose tissue derived hormone leptin together with other adipocytokines affect insulin sensitivity and is accepted to play a role in pathogenesis of obesity-related disorders [9]. The close relationship of leptin with adipose tissue and fat stores of the body suggests its involvement in the etiology and pathogenesis of NAFLD. However, there have been conflicting reports regarding the plausible role of circulating leptin and insulinemic indices in the pathogenesis of NAFLD.

The mechanisms for the gender-related differences in body fat distribution, as well as the interaction between gender and adipose tissue derived novel adipokines with respect to NAFLD, are largely unknown. The findings of the present study show that the female prediabetic subjects had significantly higher levels of serum leptin that lead to the pathogenesis of NAFLD. This is in line with other study by Huang et al that elevated serum leptin seems to be a feature of steatosis, and serum leptin seems to increase as hepatocyte steatosis develops [8]. Apiratpracha et al showed a significant higher level of body fat or subcutaneous fat than men subjects to account for the significant positive correlation between BMI in women with NAFLD and serum levels of leptin [17]. In accordance with many previous papers, serum concentrations of leptin in women were substantially higher than in men. Leptin is exclusively expressed in adipose tissue and secreted from white adipose cells. The difference is, therefore, in part, due to the higher percentage body fat in women. Such a gender difference in serum leptin levels has been reported previously [8,18,19]. The well-known gender difference in adipose tissue distribution may account for some of the difference in leptin concentration between men and women [18]. They also, added that the lower leptin levels observed in men could be due to elevated androgen concentrations [5]. Besides the increased concentration of serum leptin in female adults among South Asian, its concentration also increased with increasing age among the female children of Europe and USA who are more likely to develop NAFLD [19,20].

In the present study, NAFLD was approximately 1.3 times more prevalent in males than females which is in line with other reports [20–22]; this is in contrast by Ayonrinde et al where NAFLD to be more prevalent in females than males counterparts [23]. Using a raised ALT level as a surrogate marker of NAFLD and male subjects had higher levels of ALT within normal range compared to female subjects (Table 1). Adipose tissue distribution according to gender plays important role in the development of NAFLD because various soluble mediators (adipocytokines) are derived from fat cells and plays central role in insulin action. Though subcutaneous adipose tissue (SAT) is similar in both gender with NAFLD but male store more visceral adipose tissue (VAT) compared to female. VAT stimulates the inflammatory cells to release inflammatory cytokines such as interleukin-6 (IL-6) and tumor necrosis factor α. Moreover, free fatty acid (FFA) release by the VAT is higher in men than in women, indicating a greater lipolysis rates and deposition of excess FFA in the liver resulting NAFLD. This suggests that male subjects are more likely to develop liver disease than female subjects.

The present study also revealed a higher level of insulin resistance and reduced level of insulin secretion among the NAFLD group of both genders. NAFLD is considered the hepatic component of the metabolic syndrome and insulinemic status represents its pathophysiological hallmark. Insulin resistance in NAFLD is characterized by reduced whole-body, hepatic, and adipose tissue insulin sensitivity. The mechanism(s) underlying the accumulation of fat in the liver may include excess dietary fat, increased delivery of free fatty acids to the liver, inadequate fatty acid oxidation, and increased de novo lipogenesis. Insulin resistance is often associated with chronic low-grade inflammation, and numerous mediators released from immune cells and adipocytes may contribute liver damage and liver disease progression. Furthermore, accumulation of intra abdominal fat has also been positively correlated with liver fat [24] and hepatic insulin resistance in both men and women [25].

Leptin also had significant positive correlation with fasting and postprandial insulin, HOMA%B and HOMA-IR and significant negative correlation with HOMA%S only in male NAFLD subjects. In fact, several studies have demonstrated a significant positive correlation between plasma leptin and fasting insulin levels that is independent of body adiposity [26]. Hattori et al found that in males, the relationship between serum leptin levels and the insulin resistance was not affected by the extent of glucose intolerance [27]. Lichnovská et al reported that in men the significance of correlations between serum leptin and insulin resistance was high and approximately the same in both the control and hyperlipemic groups, because the values of insulin resistance as well as serum leptin have nearly doubled in hyperlipemic in relation to control groups [28].

Multiple regression analysis affirmed the association of serum leptin and insulinemic indices with NAFLD in both genders. In NAFLD group, only male subjects showed significant positive association with HOMA-IR after adjusting the effects of BMI, HbA_1C_, HOMA%S and HOMA%B. The underlying mechanism for this association is that the NAFLD is strongly associated with both hepatic and adipose tissue insulin resistance as well as reduced whole-body insulin sensitivity [29]. Additionally, subjects with NAFLD exhibit a defect in insulin suppression of free fatty acids (FFA), in keeping with insulin resistance at the level of the adipocyte [30]. This explanation is mainly based on the effect of insulin on hepatic fat metabolism. In this study, an elevation of fasting serum insulin level and insulin resistance in NAFLD subjects, in comparison to the controls, supports the latter suggestion. In addition, Nowak et al. found that leptin may antagonize some functions of insulin by the attenuation of insulin receptor capacity in liver [31]. In contrast, Hattori et al found no statistically significant difference in the slope of the regression of HOMA-IR for the serum level of leptin between diabetic and non-diabetic subjects [27]. de Courten et al, Schwartz et al and Kennedy et al reported that the relationship between serum levels of leptin and serum levels of insulin or insulin resistance was not affected by the extent of glucose intolerance, and that there was no significant difference in correlation between the type 2 DM and normal control subjects, which is inconsistent with our results [32–34].

Our data suggest that adiposity mediated a proportion of the association between leptin and insulinemic indices in both genders and between leptin and NAFLD only in men. The finding was inconsistent with a simple antisteatotic effect of leptin, which assumes that serum leptin values correlate with leptin biological activity in hepatocytes. There are two most likely explanations for the above finding. Firstly, leptin is inextricably related to insulin resistance. It has been suggested that leptin may contribute to hepatic steatosis by promoting insulin resistance and by altering insulin signaling in hepatocytes, so as to promote increased intracellular fatty acids. Moreover, at a later stage, leptin may cause hepatic steatosis to turn into steatohepatitis by amplifying selected proinflammatory responses [29]. From binary logistic regression analysis, HOMA%B, HOMA-IR and leptin were found to be significant determinants of NAFLD only in female prediabetes. One possible explanation for the gender difference is different distributions of fat mass by gender, e.g. more abdominal visceral adipose tissue in male and more subcutaneous adipose tissue mass in female [18,24,25]. An alternative explanation for the gender difference is that leptin may have differential effects in male and female.

In conclusion, we evaluated the gender specific relationship between serum leptin levels and insulinemic indices with NAFLD among Bangladeshi prediabetic subjects. Our study results revealed that 1) In male prediabetic subjects, insulin resistance was independently associated with serum leptin levels irrespective of adiposity and glycemic status. 2) Circulating levels of serum leptin is increased in female as compared to the male NAFLD subjects which is accompanied by pancreatic beta cell dysfunction and insulin resistance in the prediabetic subjects. 3) The relationship between the serum leptin levels, pancreatic beta cell dysfunction and the insulin resistance with NAFLD in female subjects was not affected by the degree of adiposity.

## Supporting Information

S1 Data Minimal dataset for the findings presented in this paper. (XLS)
